# A Novel Ontology Approach to Support Design for Reliability considering Environmental Effects

**DOI:** 10.1155/2015/734984

**Published:** 2015-03-04

**Authors:** Bo Sun, Yu Li, Tianyuan Ye, Yi Ren

**Affiliations:** School of Reliability and Systems Engineering, Beihang University, Beijing 100191, China

## Abstract

Environmental effects are not considered sufficiently in product design. Reliability problems caused by environmental effects are very prominent. This paper proposes a method to apply ontology approach in product design. During product reliability design and analysis, environmental effects knowledge reusing is achieved. First, the relationship of environmental effects and product reliability is analyzed. Then environmental effects ontology to describe environmental effects domain knowledge is designed. Related concepts of environmental effects are formally defined by using the ontology approach. This model can be applied to arrange environmental effects knowledge in different environments. Finally, rubber seals used in the subhumid acid rain environment are taken as an example to illustrate ontological model application on reliability design and analysis.

## 1. Introduction

Environmental conditions around military products in active service are harsh. Environmental effects have become considerable prominent. The majority of aircraft aluminous structures showed surface protective coating aging and corrosion phenomenon, such as flaking.

Many researches about impact of environmental effects on product reliability have been carried out. Melchers [1] studied erosion impact on the reliability of steel caused by the coastal environmental loads. Soares et al. [2] studied the effects of relative humidity and chlorides on the corrosion behaviour of ship steel structures and proposed a new corrosion wastage model. Dan et al. [3] studied the effects of atmosphere environmental factors on the corrosion of aluminum and aluminum alloys. Effects of environmental factors on polymer materials have also been researched extensively [4–6]. In these researches, a large amount of data, information, and experience are obtained. However, for product design, especially considering the design for reliability, these results are not applied well.

Ontological method of knowledge engineering can effectively solve problem of knowledge sharing and reusing. Environmental effects knowledge cannot achieve systematic, standardized, and repetitive applications. Gruber [7] created an early informal definition that described an ontology as “an explicit specification of a conceptualization” in 1993. Nico [8] proposed that an ontology was “a formal specification of a shared conceptualization” in 1997. Years later, Studer et al. [9] described an ontology as “a formal, explicit specification of a shared conceptualization.” Already, ontologies have been implemented in various scientific fields. Spackman et al. [10] tried to classify all the terminology and development of structured vocabularies for health care into an ontology. Yoo and No [11] established ontology-based economics knowledge sharing system. Zhang et al. [12] studied ontology-based semantic retrieval for engineering domain knowledge. Ontology models developed in engineering are focusing on developing new models into ontologies. There are both common ontology [13–15] in engineering applications and domain ontology used to describe a particular field. Various domain ontology types are proposed, including engineering design ontology [16], FMEA ontology [17], and failure ontology [18]. However, these ontological models do not achieve reasonable integration of environmental effects and reliability.

To solve the above problems, this paper uses the ontology approach to research environmental effects knowledge. By analyzing the relationship between the product reliability and environmental effects, environmental effects ontological model for reliability design and analysis was established. In the hydraulic system, the rubber seal is an important auxiliary component. Rubber seal failure can cause leakage of hydraulic system, particularly, making equipment failure. Therefore, in the design process of hydraulic system, the design and analysis of rubber seal are also important aspects. Finally, the rubber seals used in the hydraulic system were taken, for example, to demonstrate the application of environmental effects ontological model in knowledge representation.

## 2. Environmental Effects and Reliability of Product

Environmental effects were the result that performance and functionality of product were changed by physical and chemical action of environmental loads. Environmental load and product are the core elements environmental effects. Environmental loads act on the product and change product performance and functionality. It is possible to obtain influence rule of environmental factors by observing and researching change of characteristics. Environmental effects can concretely be attributed to three categories, namely, changes in mechanical properties (CMP), electrical properties (CEP), and chemical properties (CCP). As shown in Table 1, typical environmental effects of atmosphere environmental loads are briefly summarized and listed. The same environmental load reflects different environmental effects on different materials. For example, salt spray impacted on metal materials mainly caused the corrosion effect and for polymer materials mainly manifested in the physical damage.

**Figure 1:**
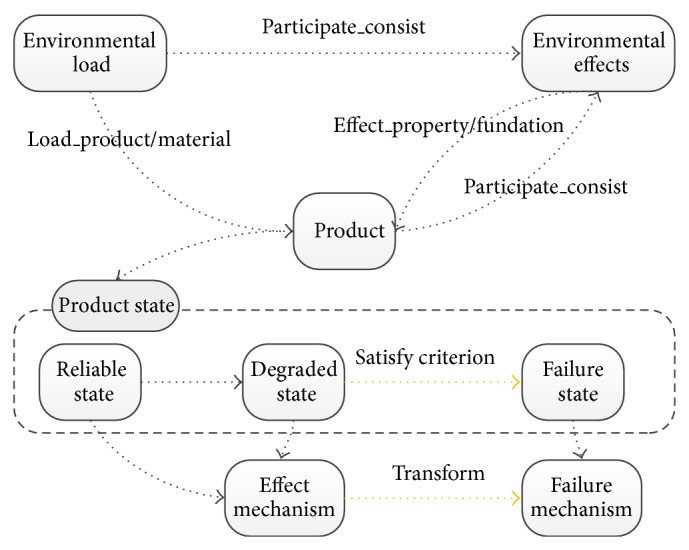
Relationships among environment effects, product, and reliability.

Environmental effects are more important factors which affect product reliability. From production, packaging, transportation, handling, storage, until the site use and protection stage, products will withstand various environmental effects. Environmental effects can cause product performance degradation, functional demotion, physical damage, and even permanent loss of function and other issues. The specific relationship between environmental effects and reliability of products is shown in Figure 1. Environmental load acts on the product that will change product state and produce environmental effects. In the beginning of action, the product can still work in a reliable state. With the passage of time, product performance degrades. Then the product becomes in degraded state. When the product continues to degrade and meets the failure criterion, the product fails; then the product is in a failure state. Effect mechanism describes the root cause of exerting environmental effects. There is a relevancy between effect mechanism and failure mechanism. When the product changes under the environment effects and reaches a predefined failure criterion, the product fails. Then effects mechanism becomes the failure mechanism. In addition, some environmental effects may form a protective impact on product, thus improving product reliability. Therefore, the advantage should be used in product design.

## 3. Ontology-Based Environmental Effects Modeling

### 3.1. Basic Definitions

According to Borst's definition [8], ontology is a formal specification of a shared conceptualization. Concepts and relationships among ontology concepts are two key elements in ontology [19]. These two elements are applied to build ontological model for domain knowledge. The basic definitions of environmental effects ontology modeling are showed as follows.

**Definition 1 (product element [18]).**

That is, a physical component of product is contained in the system without considering of the inside design in product design, such as components, general structure, and materials. Product is represented by a set of product elements. Where *c*_*i*_ is product element,

(1) C=c1,c2,…,cn ∣ ∀ci∃cj⊂ci i,j=1,2,…,n.

**Definition 2 (environmental load).**

That is, a fundamental part which owns independent variation and nature differed from the others. Where *e*_*i*_ is environmental load,

(2) E=e1,e2,…,en ∣ ∀ei∃ej⊂ei i,j=1,2,…,n.

**Definition 3 (environmental effects).**

Environmental effects were the result that performance and functionality of product were changed by physical and chemical interaction between the product and the environmental load. Moreover, environmental effects are not only works of single environmental load but also loads of multienvironment interactions. Therefore, Cartesian products of the power set of environmental loads and product elements set represented the elements involved in the process. Consider

(3) $$\begin{array}{c} P\left(E\right)=\left\{X\;|\;X\subset E\right\}\\ S=C\times P\left(E\right)=\left\{\left(c,X\right)\;|\;c\in C\wedge X\in P\left(E\right)\right\}, \end{array}$$

where *X* is a subset of environmental factor set (*E*).

**Definition 4 (environmental mode).**

That is, one region's environmental loads with unique variation characteristics are different from the others. Variations of environmental loads are different in different environment. In this study, environments which are related to the life cycle of product are just considered and one environment can be described by using a limited number of environmental loads. Each environmental load varies with times, longitude, and latitude change. Consider

(4) M=Le1,Le2,…,Len ∣ Lei=fa,l1,l2i=1,2,…,n,

where *L*_*ei*_ = *f*(*a*, *l*_1_, *l*_2_) is the general mathematical relationship that environmental loads change at different altitude (*a*), longitude (*l*_1_), and latitude (*l*_2_) is environmental mode.

### 3.2. Ontology Model

There are four kinds of relations of ontology [20], that is, part-of, kind-of, instance-of, and attribute-of, which are used to describe the interaction between the concepts of knowledge field. Part-of expresses relationship between part and whole; kind-of expresses relationship of inheriting between concepts; instance-of expresses relationship between instance and concept; attribute-of expresses that a certain concept is an attribute of another concept.

**Figure 2:**
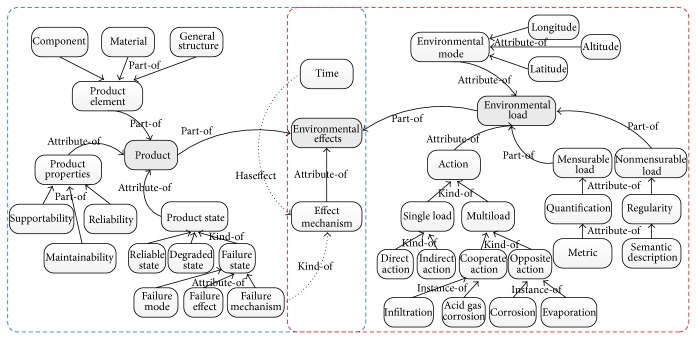
Ontological model of environmental effects.

As shown in Figure 2, environmental effects are determined by the product and the environmental loads. Therefore, environmental effects ontology consists of product subontology and environmental load subontology, specifically shown in Figure 2. Product subontology consists of product element, product state, and product property. These three concepts also have their own subconcepts.

Environmental load subontology consists of environment mode, action, and load description. There are many situations with interaction of multiloads for environmental effects. Various environmental loads would have mutual influence when they are acting on a product. This influence may be positive or negative correlation. Therefore, multiple load action is divided into synergetic action and contrary action. For example, when a thin film exists on the metal surfaces, rising temperature will increase the activation energy of a chemical reaction. And humidity load and temperature load synergistically promote corrosion conduct.

After completing construction of environmental effects ontology, environmental effects knowledge representation can be implemented according to the ontology logic. This method can make knowledge form a standardized knowledge network. Under the auxiliary of environmental effects ontology, designer can obtain more knowledge based on known information. Then new knowledge can be used for reliability design and analysis and to solve the problem that environmental effects are considered insufficiently.

## 4. Application of Environmental Effects Ontology in Design for Reliability

In order to research product reliability, life-cycle profile and mission profile of product must be determined. According to life-cycle profile, environment involved in the various stages of product life cycle can be obtained. To take advantage of environmental effects, ontology can obtain the environmental information of different environments and use the information to improve product reliability.

**Figure 3:**
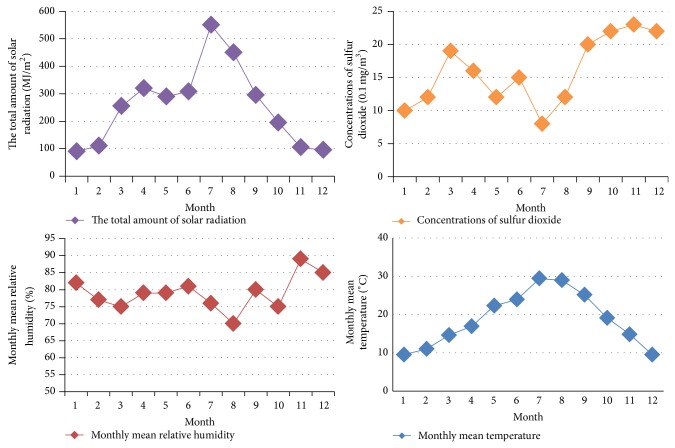
Environmental loads variation of subhumid acid rain climate.

**Figure 4:**
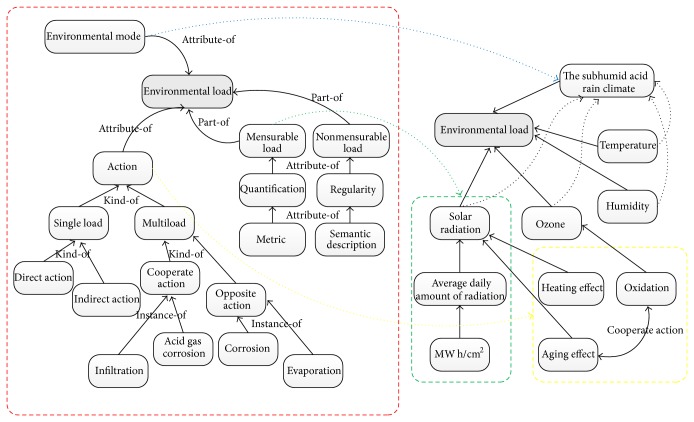
Environmental load ontology of subhumid acid rain climate.

This paper selects a typical subhumid climate of acid rain as an example to illustrate the application of environmental effects ontology. High temperature, high humidity, and heavy rain are the main climatic features. According to Figure 3, this climate zone has year-round mild climate, moist air, and abundant rainfall. The average temperature is 18.3°C. The average relative humidity is 81%. There are more than 200 days all years when relative humidity is over 80%. Annual amount of radiation in the region reached 3000 MJ, where 75% of the total amount of radiation is in summer. In the region, high temperature, high humidity, acid rain, and high levels of sulfur dioxide in the atmosphere are the main factors for material aging, rustiness, and corrosion. Based on the above analysis for subhumid acid rain climate, Figure 4 shows parts of environmental load ontology of subhumid acid rain climate.

**Figure 5:**
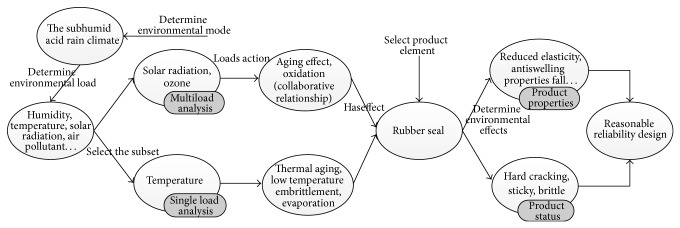
Application process of environmental effects ontology.

In the hydraulic system, the rubber seal is an important auxiliary component. Rubber seal failure can cause leakage of hydraulic system and, particularly, make equipment failure. Therefore, in the design process of hydraulic system, the design and analysis of rubber seal are also important aspects. This paper selects rubber seal as the example. Combining rubber seal, Figure 5 shows the detailed application process of environmental effects ontology model.

During design for reliability, failure modes and effects analysis (FMEA) is used widely to improve product quality and reliability. Traditional FMEA is mainly applied according to accident experience. And the environmental impact analysis is just one small side in FMEA. Therefore, this leads to insufficient consideration and unspecific analysis for environmental effects. As shown in Table 2, that is a part of rubber seal FMEA. Appling environmental effects ontology in FMEA can comprehensively and accurately analyze environmental effects on product reliability. Then we can take more targeted measures to improve product reliability design.  Table 3 shows the application of environmental effects ontology in FMEA.

Comparing Tables 2 and 3, for the same failure mode, failure causes are considered more comprehensively. Except aging, microbial mildew action is also a cause to make rubber seal sticky. Moreover, aging analysis can be more detailed for different environmental loads, such as thermal aging and hydrolysis. This makes design for reliability more targeted.

## 5. Conclusion

For the growing problem that environmental effects impact on product reliability, in this paper, from the reliability point of view, the environmental effects related research based on the ontology method was implemented. The influence of typical environmental effects on the reliability design was analyzed. The relationships among environmental effects, product, and reliability were analyzed. Ontological model of environmental effects for reliability was established. Finally, using environmental effects analysis of rubber seals as a case achieved expressing, sharing, and reusing of environmental effects knowledge in the reliability design process. Proposing ontological model of environmental effects can reduce the repeated questions of reliability design process. This paper applied the ontological model to establish preliminarily knowledge networks of subhumid acid rain climate. The next work will continue to establish more complete knowledge base and apply environmental effects knowledge in product design. In addition, the ontology approach focuses on qualitative analysis. In subsequent research, we need to apply other approaches to research quantitative analysis of environmental effects in reliability design and analysis.

## Figures and Tables

**Table 1:** Typical environmental effects of atmosphere.

Environmental load	Typical material	Classification of environmental effects	Typical environmental effects
Temperature	MM	CEP	Metal resistivity change
	PM	CMP	Material expansion and deformation
		CEP	Insulation changes

Humidity	MM	CCP	Corrosion: increase the rate of chemical reaction
		CEP	Metallic conductivity changes
	PM	CMP	Reduce lubricant performance
		CEP	Electrical short circuit

Salt spray	MM	CMP	Block: wear and tear
		CEP	Metallic conductivity changes
	PM	CMP	Damage protective coating
		CCP	Corrosion

Notes:

MM: metallic materials. PM: polymer materials.

**Table 2:** Rubber seals FMEA analysis.

Product	Function	Failure mode	Cause
Rubber seals	Seal	Hard cracking, leak	Ageing Corrosion
		Fracture, cuts uneven local	Mechanical injury

**Table 3:** Rubber seals FMEA analysis (applying environmental effects ontology).

Product	Function	Environmental load	Failure mode	Cause	Failure mechanism
Rubber seal	Seal	Salt spray, dust	Fracture, cuts uneven local	Mechanical injury	Salt enters into space between seal ring and other structures and results in wear of seal ring in the relative movement
				Ageing	
		Solar radiation, oxygen (ozone)	Hard cracking, sticky, brittle	Photooxidation aging	Rubber macromolecules decompose due to the absorption of photons, and molecular bond is oxidized under the action of oxygen
		High temperature, oxygen (ozone)		Thermal aging	Rubber macromolecules decompose due to the absorption of heat, and molecular bond is oxidized under the action of oxygen
		Precipitation, condensation		Absorbent hydrolyzed	Deliquescence of rubber molecules due to absorbing moisture
		Microbe, humidity, temperature		Mildew	In suitable environment, microorganism grows and breeds in the rubber, thereby reducing material properties
		Air pollutant	Leak, break	Corrosion	Ozone and other oxidants can oxidize rubber molecules bond
		Low pressure		Deformation	Unequal internal and external pressure make rubber seal deformation
		Ozone (with stress)		Ozone-induced cracking	Rubber seal oxidized by ozone was stressed

## References

[1] Melchers R. E. (2005). The effect of corrosion on the structural reliability of steel offshore structures. *Corrosion Science*.

[2] Soares C. G., Garbatov Y., Zayed A., Wang G. (2009). Influence of environmental factors on corrosion of ship structures in marine atmosphere. *Corrosion Science*.

[3] Dan Z., Muto I., Hara N. (2012). Effects of environmental factors on atmospheric corrosion of aluminium and its alloys under constant dew point conditions. *Corrosion Science*.

[4] Torikai A., Takeuchi A., Fueki K. (1986). The effect of temperature on the photo-degradation of polystyrene. *Polymer Degradation and Stability*.

[5] Ito M., Nagai K. (2008). Degradation issues of polymer materials used in railway field. *Polymer Degradation and Stability*.

[6] Li C., Zhang M., Miao L. (2014). Effects of environmental factors on the conversion efficiency of solar thermoelectric co-generators comprising parabola trough collectors and thermoelectric modules without evacuated tubular collector. *Energy Conversion and Management*.

[7] Gruber T. R. (1993). A translation approach to portable ontology specifications. *Knowledge Acquisition*.

[8] Nico B. W. (1997). *Construction of engineering ontologies for knowledge sharing and reuse [Ph.D. dissertation]*.

[9] Studer R., Benjamins V. R., Fensel D. (1998). Knowledge engineering: principles and methods. *Data & Knowledge Engineering*.

[10] Spackman K. A., Campbell K. E., Côté R. A. (1997). SNOMED RT: a reference terminology for health care. *Journal of the American Medical Informatics Association*.

[11] Yoo D., No S. (2014). Ontology-based economics knowledge sharing system. *Expert Systems with Applications*.

[12] Zhang X., Hou X., Chen X., Zhuang T. (2013). Ontology-based semantic retrieval for engineering domain knowledge. *Neurocomputing*.

[13] De Kleer J. (1984). How circuits work. *Artificial Intelligence*.

[14] Kitamura Y., Mizoguchi R. Ontology-based functional-knowledge modeling methodology and its deployment.

[15] Li Z., Raskin V., Ramani K. (2008). Developing engineering ontology for information retrieval. *Journal of Computing and Information Science in Engineering*.

[16] Borst P., Akkermans H., Top J. (1997). Engineering ontologies. *International Journal of Human-Computer Studies*.

[17] Ebrahimipour V., Rezaie K., Shokravi S. (2010). An ontology approach to support FMEA studies. *Expert Systems with Applications*.

[18] Ren Y., Sun B., Feng Q., Zeng S. (2011). Ontological multi-view failure modeling for IPPD. *Maintenance and Reliability*.

[19] Devedzić V. (2002). Understanding ontological engineering. *Communications of the ACM*.

[20] Huang H.-Z., Tian Z., Zuo M. J. (2005). Intelligent interactive multiobjective optimization method and its application to reliability optimization. *IIE Transactions*.

